# Supplementary material for: Abundant Recurrent Mitochondrial Mutations and Widespread Mitonuclear Epistasis in Caenorhabditis elegans
Source: Mol Biol Evol. 2025 Dec 10;42(12):msaf300. doi: 10.1093/molbev/msaf300 (PMC12690269; doi:10.1093/molbev/msaf300)
Supplement: msaf300_Supplementary_Data [file msaf300_supplementary_data.zip › Figure S7.pdf]

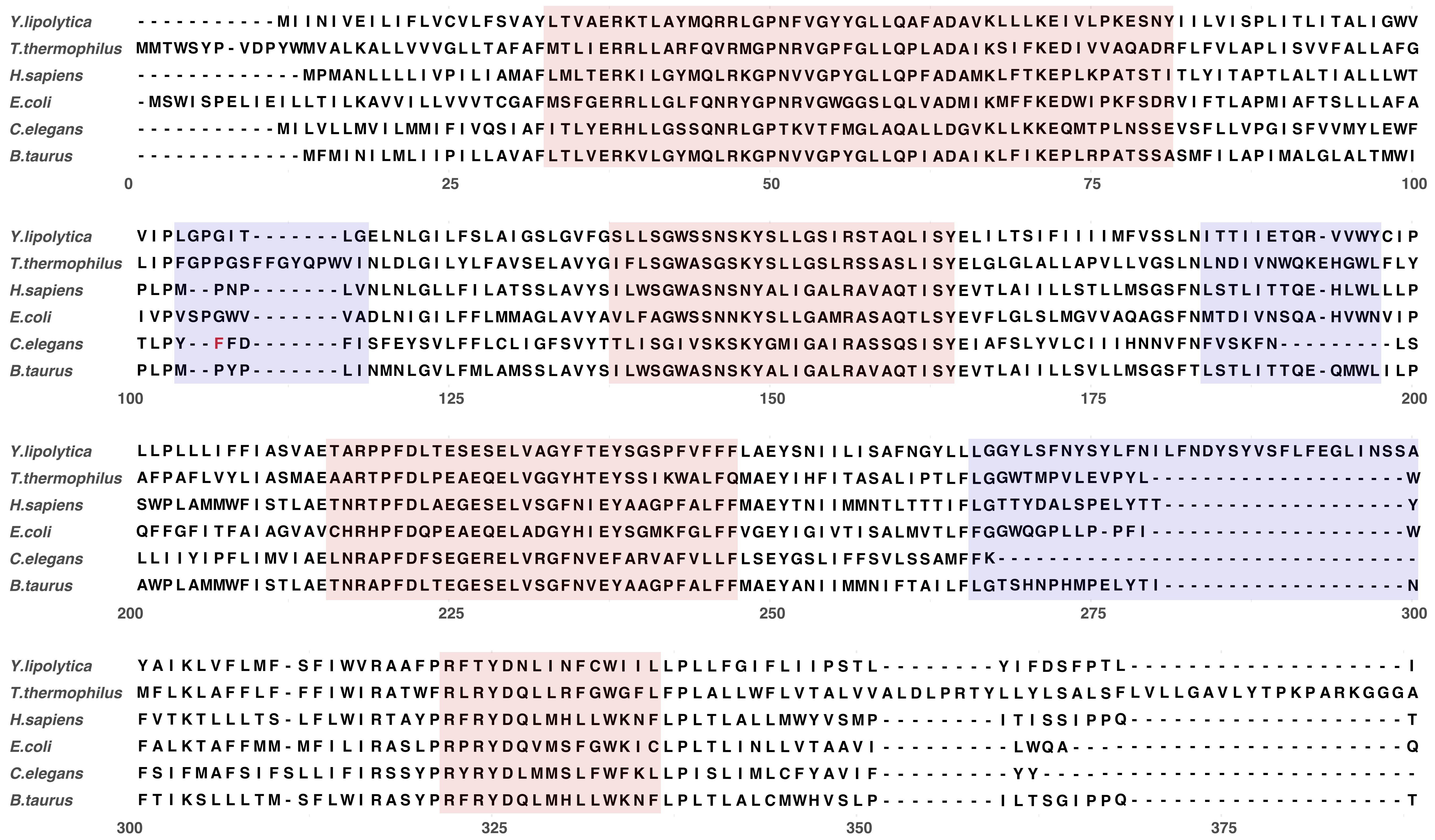
Y.lipolytica -----------MIINIVEILIFLVCVLFSVAYLTVAERKTLAYMQRRLGPNFVGYYGLLQAFADAVKLLLKEIVLPKESNYIILVISPLITLITALIGWV
T.thermophilus MMTWSYP-VDPYWMVALKALLVVVGLLTAFAFMTLIERRLLARFQVRMGPNRVGPFGLLQPLADAIKSIFKEDIVVAQADRFLFVLAPLISVVFALLAFG
H.sapiens -------------MPMANLLLLIVPILIAMAFLMLTERKILGYMQLRKGPNVVGPYGLLQPFADAMKLFTKEPLKPATSTITLYITAPTLALTIALLLWT
E.coli -MSWISPELIEILLTILKAVVILLVVVTCGAFMSFGERRLLGLFQNRYGPNRVGWGGSLQLVADMIKMFFKEDWIPKFSDRVIFTLAPMIAFTSLLLAFA
C.elegans -----------MILVLLMVILMMIFIVQSIAFITLYERHLLGSSQNRLGPTKVTFMGLAQALLDGVKLLKKEQMTPLNSSEVSFLLVPGISFVVMYLEWF
B.taurus -------------MFMINILMLIIPILLAVAFLTLVERKVLGYMQLRKGPNVVGPYGLLQPIADAIKLFIKEPLRPATSSASMFILAPIMALGLALTMWI
0 25 50 75 100
Y.lipolytica VIPLGPGIT-------LGELNLGILFSLAIGSLGVFGSLLSGWSSNSKYSLLGSIRSTAQLISYELILTSIFIIIIMFVSSLNITTIIETQR-VVWYCIP
T.thermophilus LIPFGPPGSFFGYQPWVINLDLGILYLFAVSELAVYGIFLSGWASGSKYSLLGSLRSSASLISYELGLGLALLAPVLLVGSLNLNDIVNWQKEHGWLFLY
H.sapiens PLPM--PNP-------LVNLNLGLLFILATSSLAVYSILWSGWASNSNYALIGALRAVAQTISYEVTLAIILLSTLLMSGSFNLSTLITTQE-HLWLLLP
E.coli IVPVSPGWV-------VADLNIGILFFLMMAGLAVYAVLFAGWSSNNKYSLLGAMRASAQTLSYEVFLGLSLMGVVAQAGSFNMTDIVNSQA-HVWNVIP
C.elegans TLPY--FFD-------FISFEYSVLFFLCLIGFSVYTTLISGIVSKSKYGMIGAIRASSQSISYEIAFSLYVLCIIIHNNVFNFVSKFN---------LS
B.taurus PLPM--PYP-------LINMNLGVLFMLAMSSLAVYSILWSGWASNSKYALIGALRAVAQTISYEVTLAIILLSVLLMSGSFTLSTLITTQE-QMWLILP
100 125 150 175 200
Y.lipolytica LLPLLLIFFIASVAETARPPFDLTESESELVAGYFTEYSGSPFVFFFLAEYSNIILISAFNGYLLLGGYLSFNYSYLFNILFNDYSYVSFLFEGLINSSA
T.thermophilus AFPAFLVYLIASMAEAARTPFDLPEAEQELVGGYHTEYSSIKWALFQMAEYIHFITASALIPTLFLGGWTMPVLEVPYL--------------------W
H.sapiens SWPLAMMWFISTLAETNRTPFDLAEGESELVSGFNIEYAAGPFALFFMAEYTNIIMMNTLTTTIFLGTTYDALSPELYTT-------------------Y
E.coli QFFGFITFAIAGVAVCHRHPFDQPEAEQELADGYHIEYSGMKFGLFFVGEYIGIVTISALMVTLFFGGWQGPLLP-PFI--------------------W
C.elegans LLIIYIPFLIMVIAELNRAPFDFSEGERELVRGFNVEFARVAFVLLFLSEYGSLIFFSVLSSAMFFK---------------------------------
B.taurus AWPLAMMWFISTLAETNRAPFDLTEGESELVSGFNVEYAAGPFALFFMAEYANIIMMNIFTAILFLGTSHNPHMPELYTI-------------------N
200 225 250 275 300
Y.lipolytica YAIKLVFLMF-SFIWVRAAFPRFTYDNLINFCWIILLPLLFGIFLIIPSTL--------YIFDSFPTL-------------------I
T.thermophilus MFLKLAFFLF-FFIWIRATWFRLRYDQLLRFGWGFLFPLALLWFLVTALVVALDLPRTYLLYLSALSFLVLLGAVLYTPKPARKGGGA
H.sapiens FVTKTLLLTS-LFLWIRTAYPRFRYDQLMHLLWKNFLPLTLALLMWYVSMP--------ITISSIPPQ-------------------T
E.coli FALKTAFFMM-MFILIRASLPRPRYDQVMSFGWKICLPLTLINLLVTAAVI---------LWQA-----------------------Q
C.elegans FSIFMAFSIFSLLIFIRSSYPRYRYDLMMSLFWFKLLPISLIMLCFYAVIF---------YY--------------------------
B.taurus FTIKSLLLTM-SFLWIRASYPRFRYDQLMHLLWKNFLPLTLALCMWHVSLP--------ILTSGIPPQ-------------------T
300 325 350 375
