# Supplementary material for: Abundant Recurrent Mitochondrial Mutations and Widespread Mitonuclear Epistasis in Caenorhabditis elegans
Source: Mol Biol Evol. 2025 Dec 10;42(12):msaf300. doi: 10.1093/molbev/msaf300 (PMC12690269; doi:10.1093/molbev/msaf300)
Supplement: msaf300_Supplementary_Data [file msaf300_supplementary_data.zip › Figure S8.pdf]

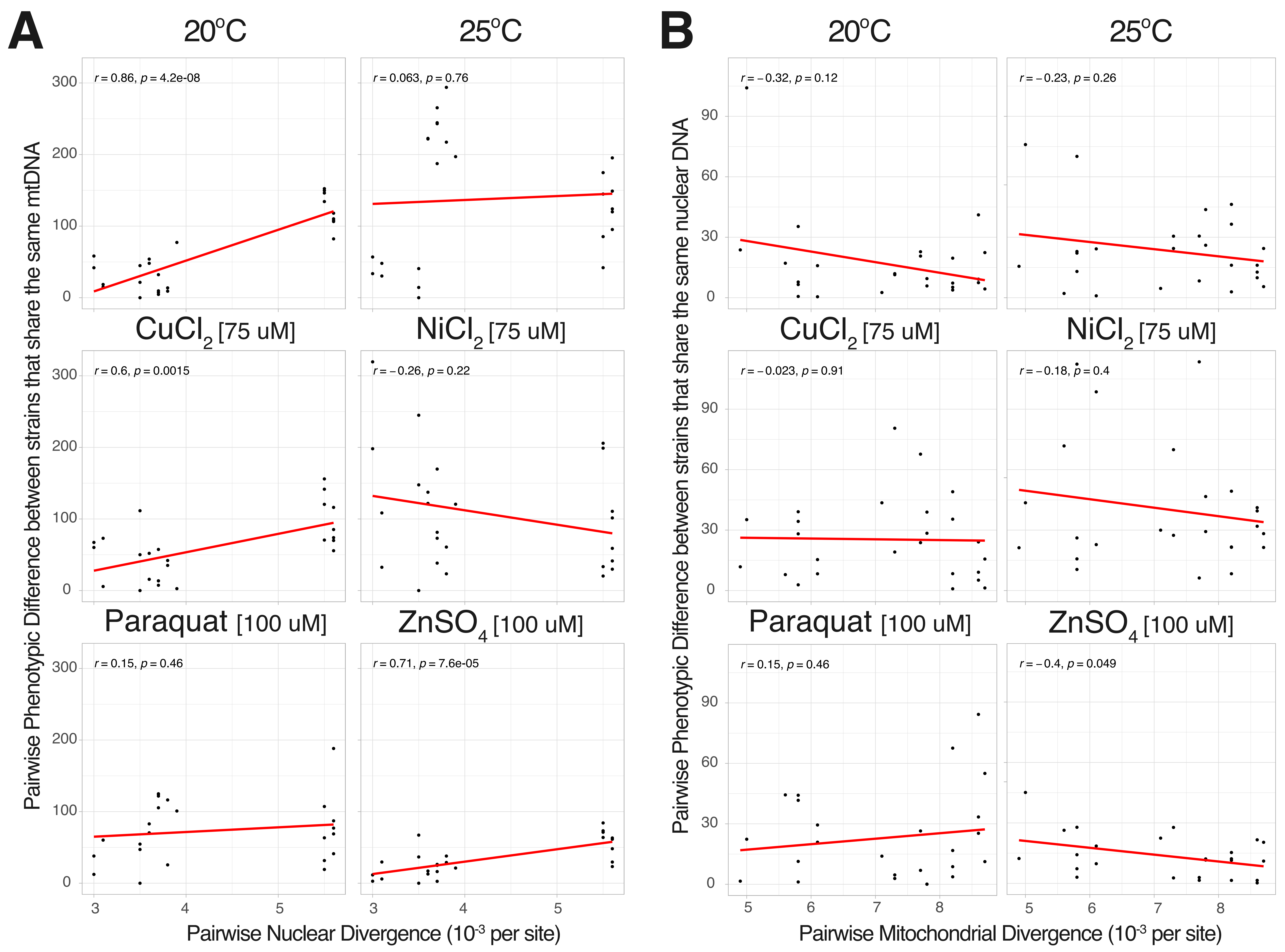

A
B
20°C
25°C
CuCl2 [75 uM]
NiCl2 [75 uM]
Paraquat [100 uM]
ZnSO4 [100 uM]
r = 0.86, p = 4.2e-08
r = 0.063, p = 0.76
r = 0.6, p = 0.0015
r = −0.26, p = 0.22
r = 0.15, p = 0.46
r = 0.71, p = 7.6e-05
r = −0.32, p = 0.12
r = −0.23, p = 0.26
r = −0.023, p = 0.91
r = −0.18, p = 0.4
r = 0.15, p = 0.46
r = −0.4, p = 0.049
Pairwise Phenotypic Difference between strains that share the same mtDNA
Pairwise Nuclear Divergence (10-3 per site)
Pairwise Phenotypic Difference between strains that share the same nuclear DNA
Pairwise Mitochondrial Divergence (10-3 per site)
