# Supplementary material for: Abundant Recurrent Mitochondrial Mutations and Widespread Mitonuclear Epistasis in Caenorhabditis elegans
Source: Mol Biol Evol. 2025 Dec 10;42(12):msaf300. doi: 10.1093/molbev/msaf300 (PMC12690269; doi:10.1093/molbev/msaf300)
Supplement: msaf300_Supplementary_Data [file msaf300_supplementary_data.zip › supplementarydoc.docx]

**Figure S1. *Caenorhabditis inopinata* mitochondrial genome is substantially divergent from *Caenorhabditis elegans* mitochondrial genome. Maximum Likelihood Phylogenetic tree** based on alignment of concatenated amino acid sequences from *C. inopinata* and 367 *C. elegans* isotypes.

**Figure S2.** **Phylogenetic distribution of mitochondrial functional variants**. **A.** Number of functional changes (missense, rRNA, and tRNA) on the internal branches that connect mitochondrial haplogroups. **B-O** **Functional changes across mitochondrial protein-coding genes**. Branches exhibiting state transitions (mutations) are highlighted in red, while those with no detected changes remain in grey. The thickness of each branch is scaled to reflect the number of mutations, with thicker branches indicating a higher number of mutations.

**Figure S3.** **A. Distribution of the most recurrent mutation (mtDNA:2042) on the mitochondrial phylogeny.** Strains carrying alternative alleles are mapped onto the tree, with red indicating strains carrying the first alternative allele A (94F>94I) and blue representing strain with the second alternative allele C (94F>94L). **B. The 94^th^ residue is highly conserved within *Caenorhabditis* genus.** Shown are amino acid residues from positions 50 to 100, with the 94^th^ residue indicated by a black arrow. Phylogenetic tree was generated based on whole gene alignment using mafft online server (Katoh, et al. 2017). Gene tree does not recapitulate species tree. **C. Amino acid conservation analysis of the mitochondrial peptide nduo-1 across a broader evolutionary scale.** Shown are amino acid residues from positions 50 to 100, with the 94^th^ residue indicated by a black arrow. Conservation analysis was performed using ConSurf server, which identified homologous sequences from UNIREF90 using the HMMER search algorithm. Pairwise identity thresholds were set to a maximum of 95% and a minimum of 80%. A multiple sequence alignment was built using MAFFT, and 12 representative sequences were selected to the final analysis. Phylogeny does not reflect species phylogeny. Conservation scores range from 1 (highly variable, teal) to 9 (highly conserved, purple) (Yariv, et al. 2023).

**Figure S4. Genome-wide visualization of allele-specific 25-mer support across nuclear and mitochondrial chromosomes for six strains.** Each vertical line represents read support for a unique 25-mer centered on a diagnostic SNP distinguishing ECA1229 (red) and ECA1493 (blue) alleles. Line thickness indicates the number of reads matching that allele’s 25-mer at each genomic position. **Top row:** wild isolates ECA1229 and ECA1493 with native mitonuclear genomes. **Middle row:** reconstructed strains (QG4499, QG4454) with matched mitonuclear genotypes via GPR-1–mediated mitochondrial transfer. **Bottom row:** cybrids (QG4505, QG4448) with swapped mitochondrial genomes. Scattered loci with both red and blue support likely reflect sequencing or mapping noise.

**Figure S5. Interaction plots for strain pairs showing significant mitonuclear epistasis induced by mitochondrial swaps in all conditions.** Each plot includes two matched mitonuclear combinations and two mismatched mitonuclear combinations. The shown quartets only represent cases where significant mitonuclear epistasis was detected (ANOVA, P < 0.05).

**Figure S6.** Relative phenotypic changes strains with mismatched mitonuclear genotypes to matched mitonuclear genotypes within the same nuclear background. Lines connecting the matched (reconstituted parental combination) and synthetic mitonuclear are colored to indicate a statistically significant reduction (red), increase (black), or less than 5% change (gray). See **Table S9** for statistics.

**Figure S7**. **Sequence alignment of the complex I subunit ND1/NDUO1/NuoH/Nqo8.** Residues belonging to the inner mitochondrial matrix or the inside ends of the predicted transmembrane helices are highlighted in red. Residues belonging to the intermembrane space or the outside of the predicted transmembrane helices are highlighted in purple. The 94^th^ codon of *C. elegans* NDUO1 is depicted in red. The alignment was carried out using mafft with default parameters. Accession numbers for sequences are (in order of appearance): CAC28089.2, AAA97945.1, CAA24026.1, CAA48367.1, AAO16387.1, CAA23997.1.

**Figure S8. Correlation between phenotypic differences and genetic divergence. A**. Cybrids sharing the same mitochondrial genome but differing in nuclear genomes, plotted against nuclear divergence. **B**. Cybrids sharing the same nuclear genome but differing in mitochondrial genomes, plotted against mitochondrial divergence.  Each point represents a cybrid–parent comparison under a given environment. Pearson’s *r* and *P* values are shown.

**Table S1. Strain information and mitochondrial haplotype classification.** Table includes strain names, isotypes, geographic coordinates (latitude and longitude), locations of isolation, collection metadata from CaeNDR (substrate, temperature, humidity), mitochondrial haplogroups, shared common haplotypes, and strains included in the 18×18 and 6×6 panels. Highlighted red cells reflect isotypes that share the same mitochondrial genotypes.

**Table S2. Intraspecific variant annotations of all mitochondrial SNPs segregating among *C. elegans* isotype reference strains**. Table includes the 1,458 mitochondrial sites that segregate among 367 C. *elegans* isotypes along with predicted amino acid changes using SNPEff (version 5.1). There are multiple rows for some sites because they segregate more than two variants.

**Table S3. Haplotype pairs differing by a single mitochondrial mutation among 367 isotypes.** Table highlights 144 single mitochondrial mutations that can be tested individually, including 63 missense mutations and 26 RNA mutations, to reveal the effects of specific mitochondrial changes. Highlighted cells reflect instances where there are single missense differences with the N2 reference genome.

**Table S4. Intra-isotype variants.** 28 mitochondrial sites that segregate amongst 832 other natural isolates that were not included in 540 isotypes.

**Table S5. Characterization of recurrent mutations on the mitochondrial phylogeny.** 86 SNPs (from the set of 472 missense or RNA-altering SNPs) that required at least 2 mutations on the tree are reported, along with the genes in which they are found, variant types, and minimum number of changes at that site required by the tree.

**Table S6. Diagnostic functional mitochondrial variants for each mitochondrial haplogroup.**  Recurrent mutations are highlighted.

**Table S7. dN/dS ratio estimated by HyPhy for the internal branches and the tip branches for each mitochondrial coding gene.**

**Table S8.** **Strain pairs showing significant mitonuclear epistasis induced by mitochondrial swaps in all conditions**. For each of the strain pair, ANOVAs comparing the full mixed effect model with a model lacking the mitonuclear (N × MT) term were evaluated. The mitonuclear term was treated as a fixed effect, while batch factor was treated as a random effect**.**

**Table S9. The effect of mitochondrial DNA swap in each nuclear background across all environments.** To determine whether the difference among strains with matched and mismatched combinations is statistically significant, ANOVAs comparing the full mixed effect model with a model lacking the Mito term were evaluated. The Mito term was treated as a fixed effect, while batch factor was treated as a random effect.

**Table S10. Accession number for ND1 amino acid sequences from *Caenorhabditis* species and the outgroup *Diploscapter coronatus.***

**Table S11. Codons that vary at multiple positions.** The table includes codon genotypes, their corresponding amino acids (based on the invertebrate mitochondrial genetic code), and the number of strains carrying each genotype. Red numbers indicate nucleotide positions with variants. The highlighted row denotes a case of SnpEff misannotation, where the change was predicted as a missense mutation, though the corresponding genotype is not observed in the population.

**Supplemental File 1**. Additional details on model parameters, rate heterogeneity, and tree statistics on intra *C. elegans* mitochondrial phylogeny

**Supplemental Files 2.** Additional details on model parameters, and statistics in all protein coding genes using Mixed Effect Model of Evolution in HyPhy.

**Supplemental File 3.** Additional details on model parameters, rate heterogeneity, and tree statistics on mitochondrially encoded amino acid phylogeny with *C. inopinata* outgroup.
