# Supplementary figures and images for: Abundant Recurrent Mitochondrial Mutations and Widespread Mitonuclear Epistasis in Caenorhabditis elegans
Source: Mol Biol Evol. 2025 Dec 10;42(12):msaf300. doi: 10.1093/molbev/msaf300 (PMC12690269; doi:10.1093/molbev/msaf300)

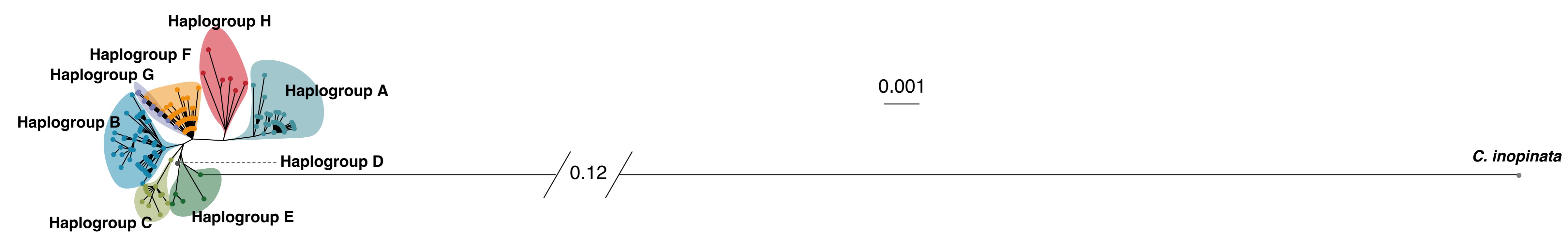

Supplement: msaf300_Supplementary_Data [file msaf300_supplementary_data.zip › Figure S1.pdf]

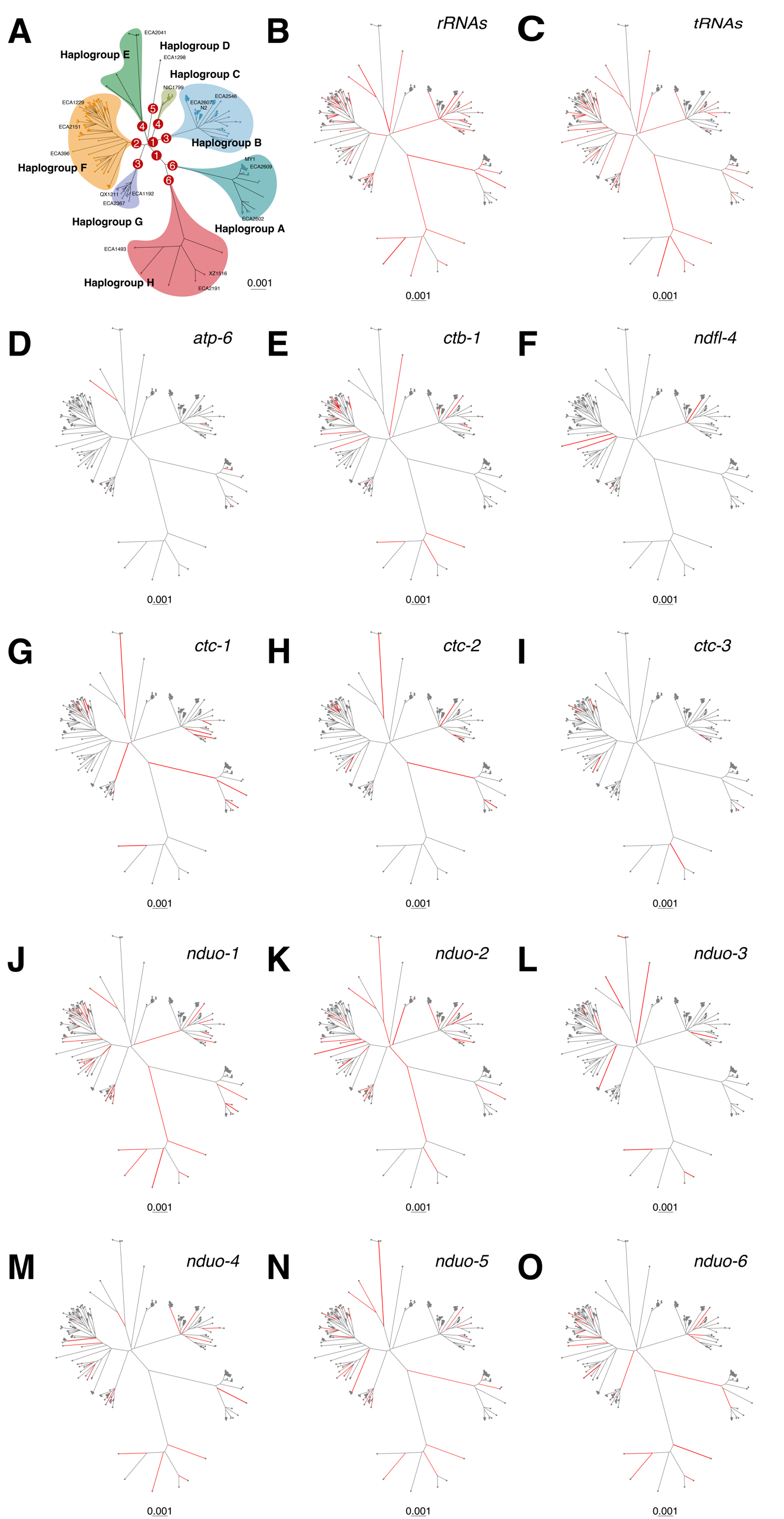

Supplement: msaf300_Supplementary_Data [file msaf300_supplementary_data.zip › Figure S2.pdf]

A

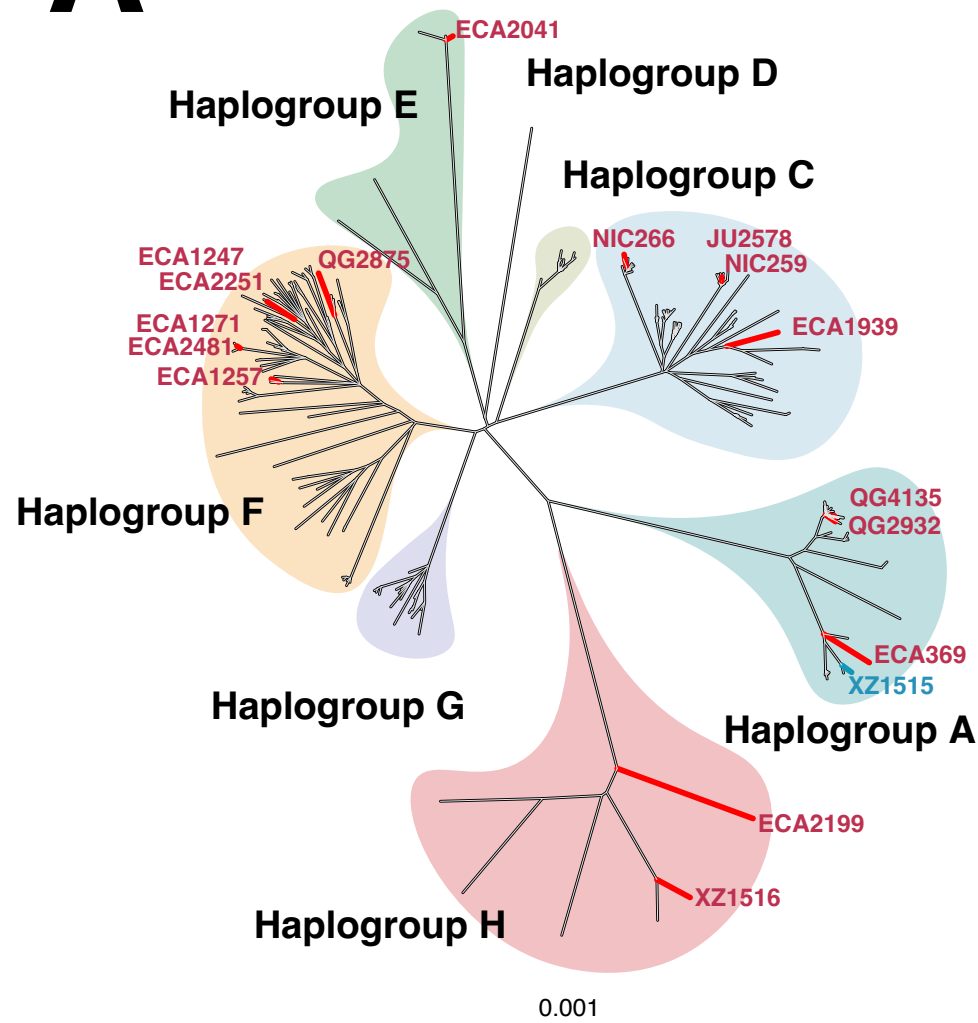

B

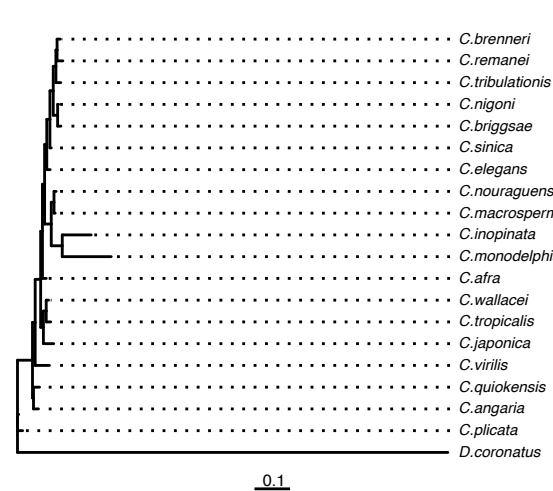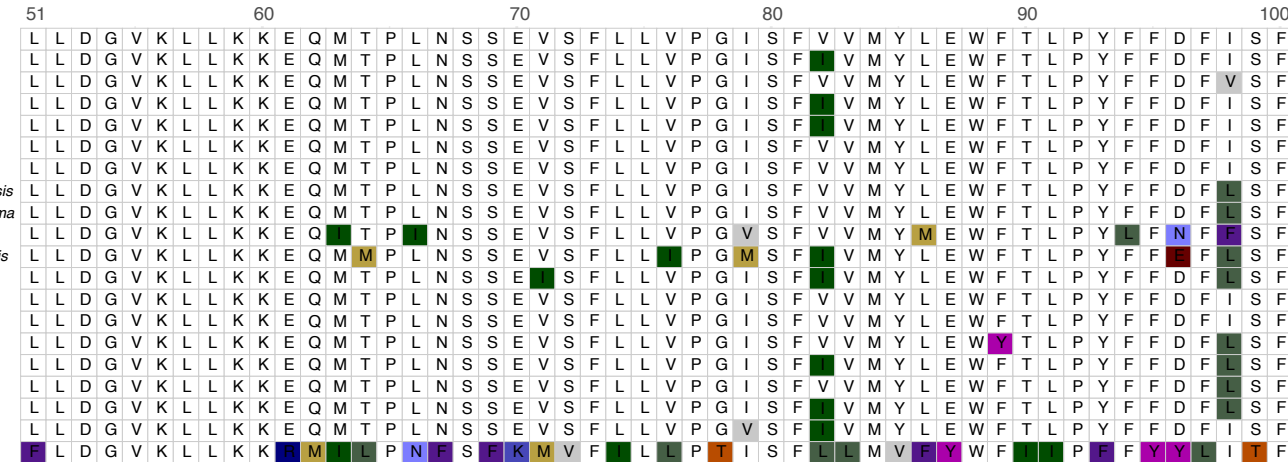

C

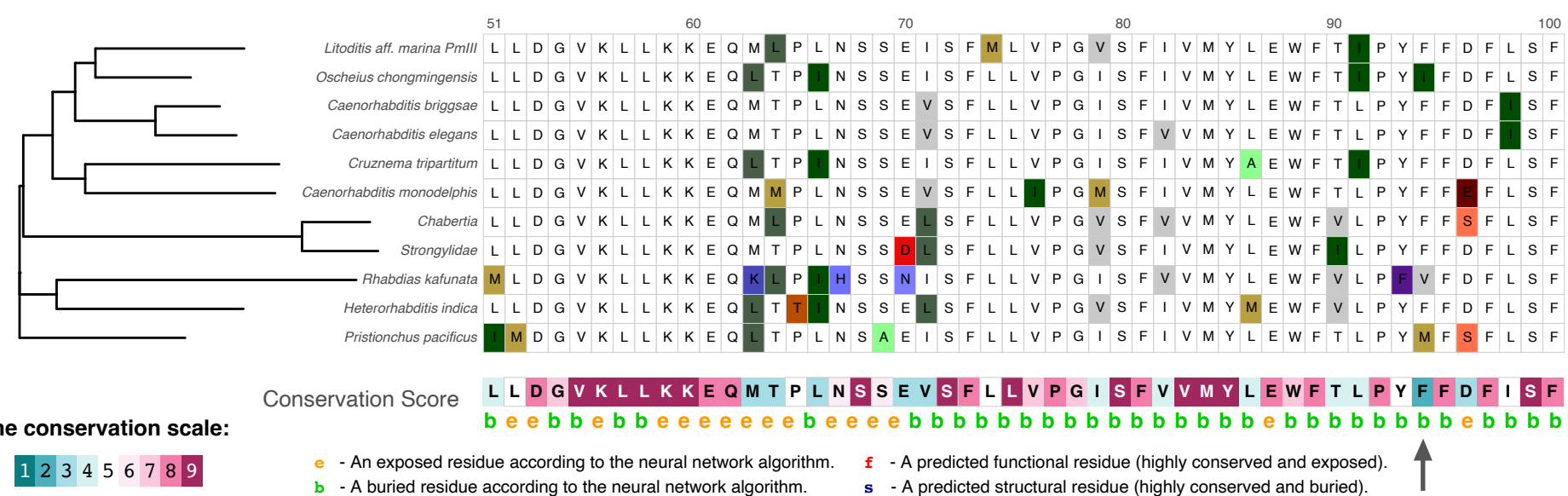

Supplement: msaf300_Supplementary_Data [file msaf300_supplementary_data.zip › Figure S3.pdf]

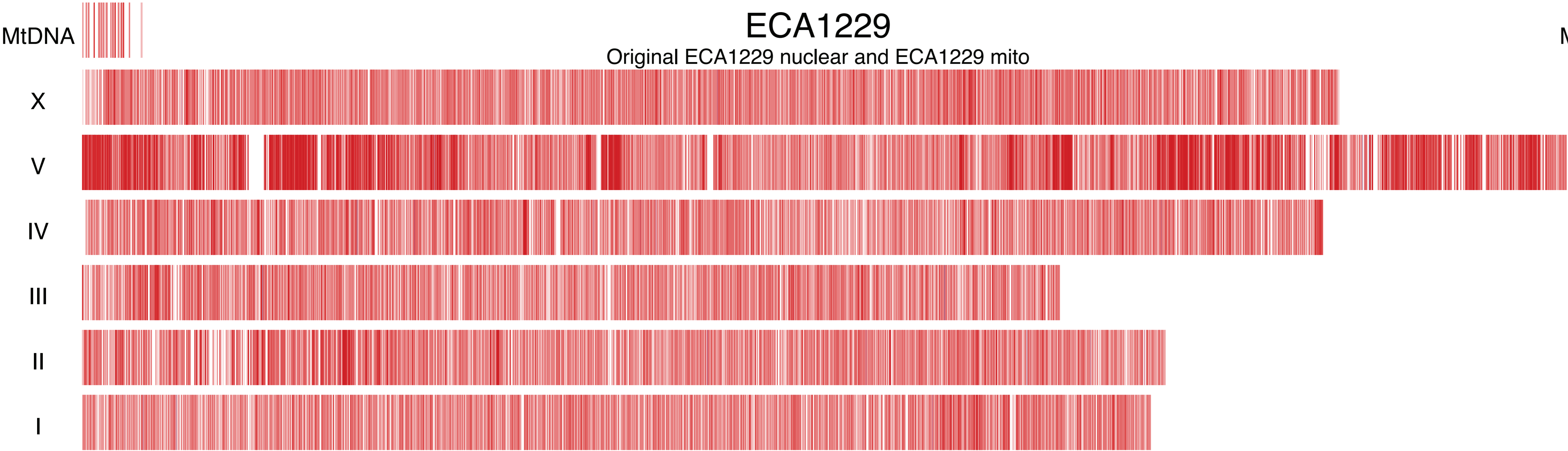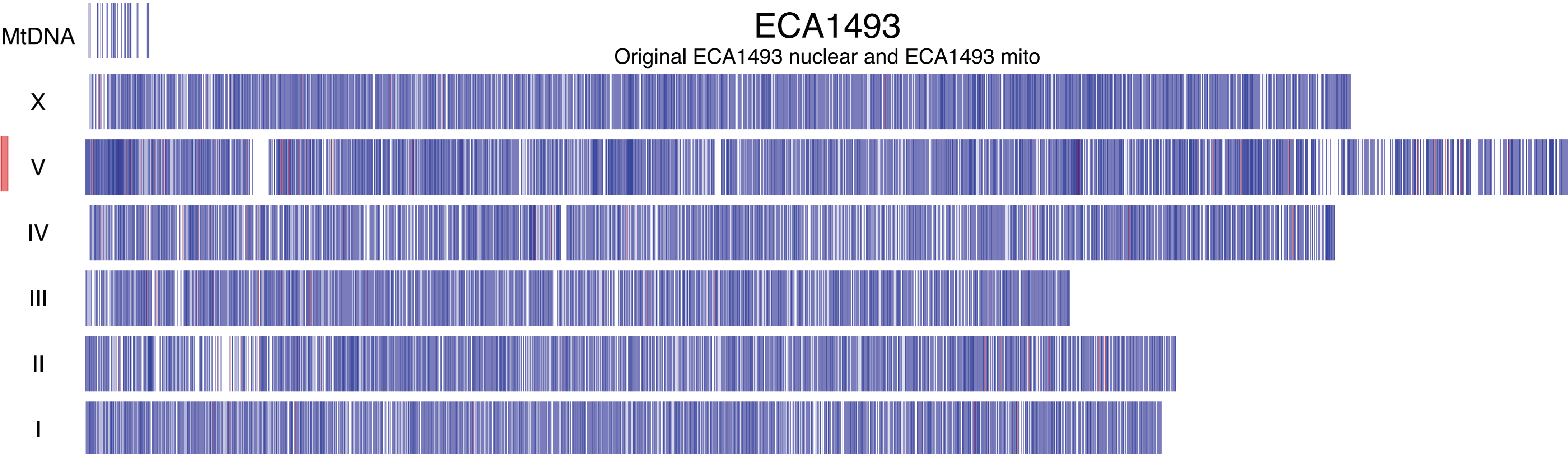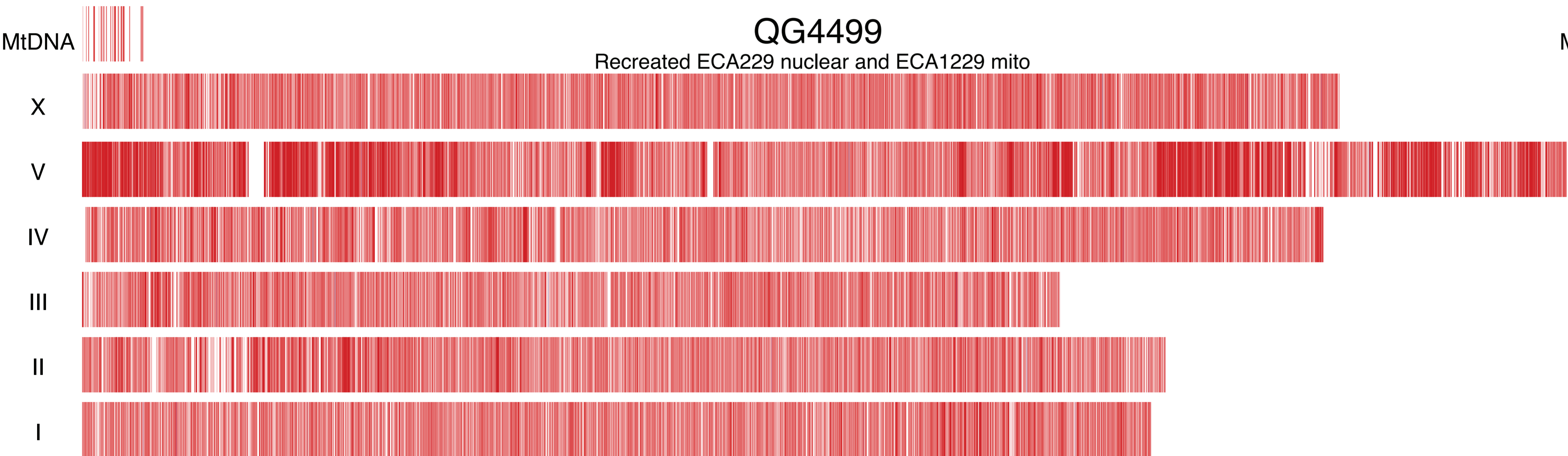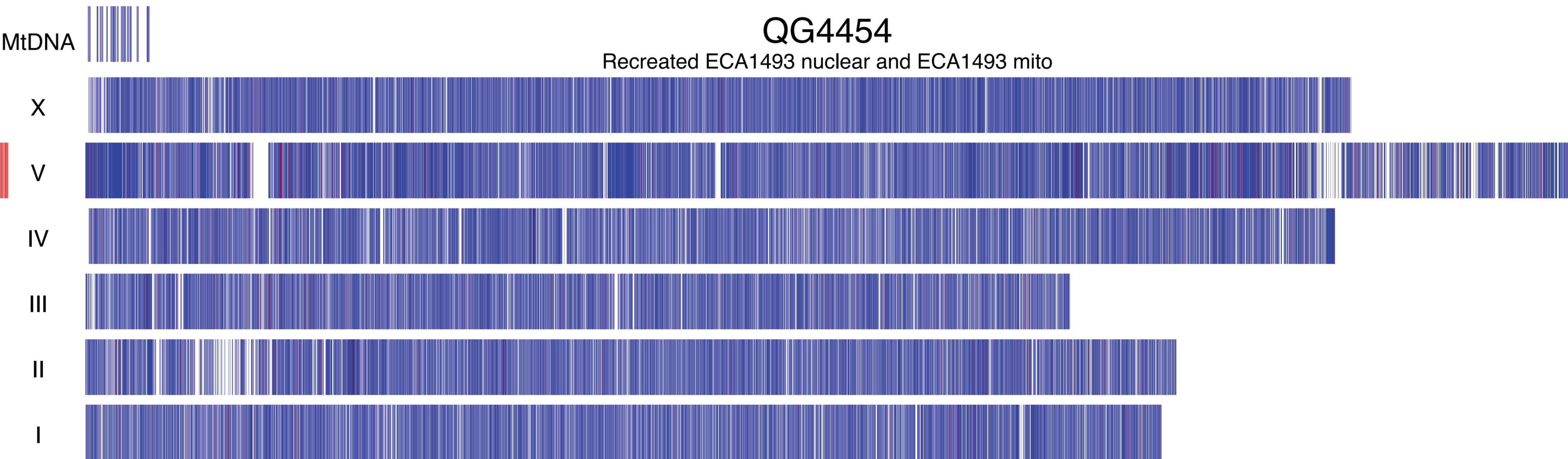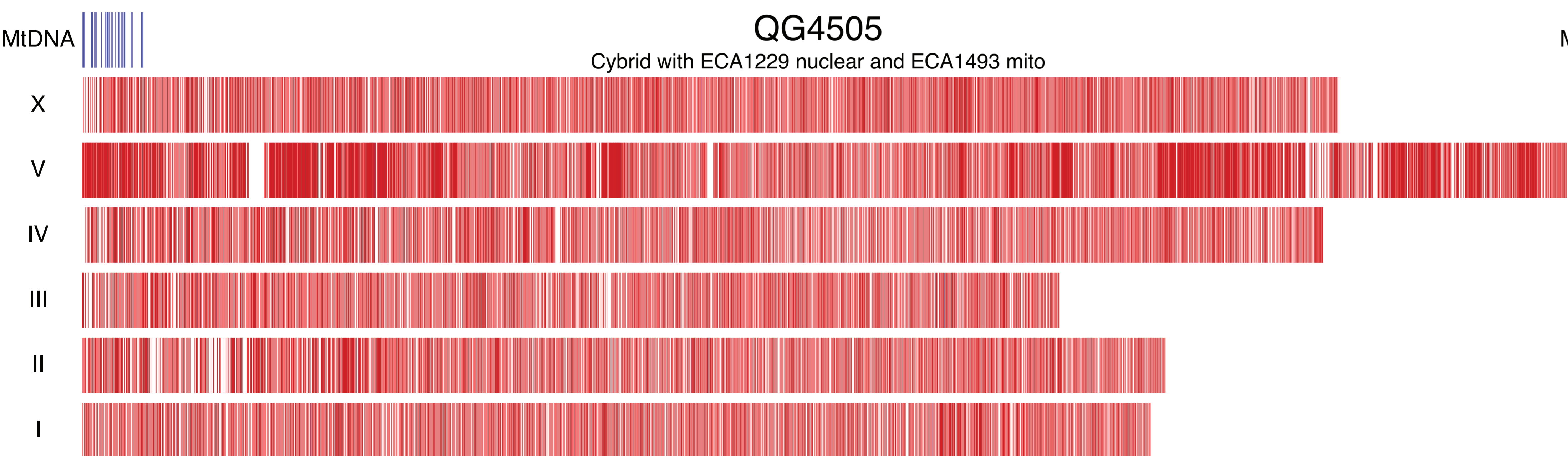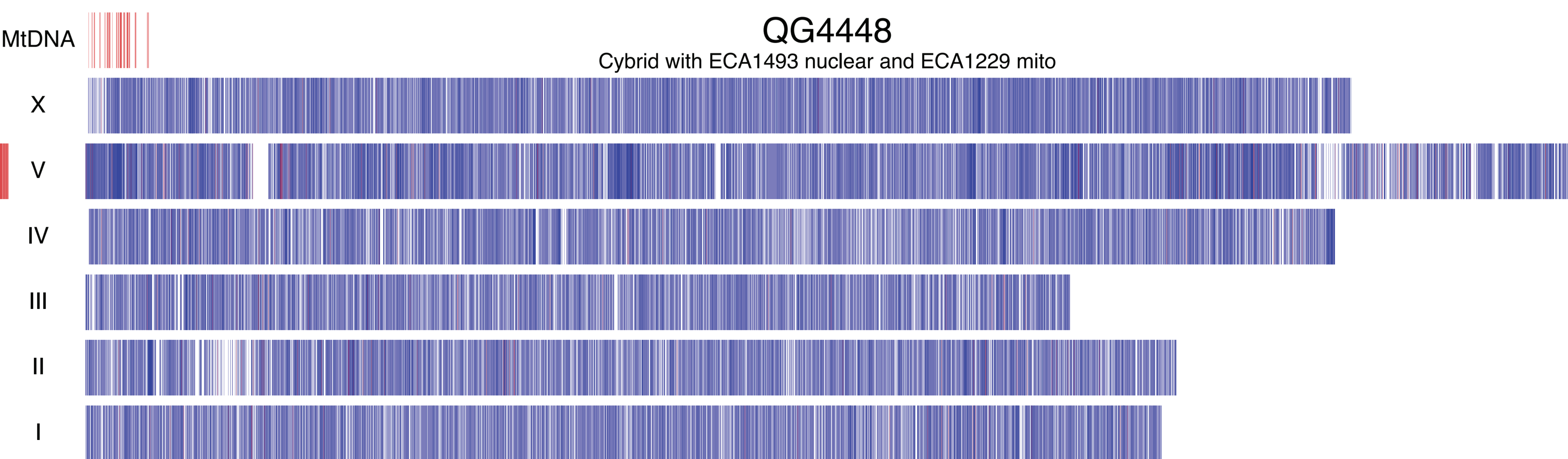

Supplement: msaf300_Supplementary_Data [file msaf300_supplementary_data.zip › Figure S4.pdf]

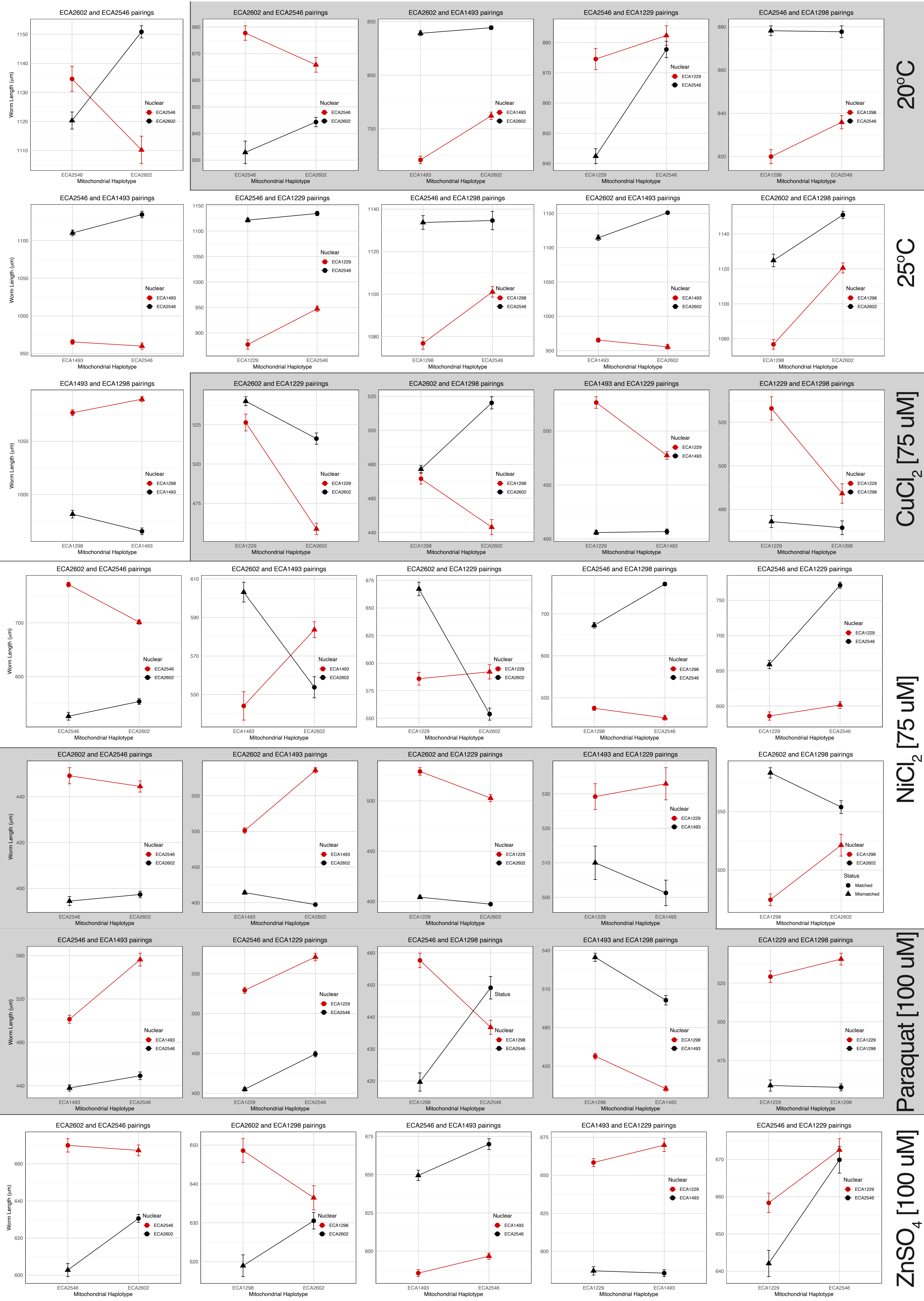

Supplement: msaf300_Supplementary_Data [file msaf300_supplementary_data.zip › Figure S5.pdf]

20°C

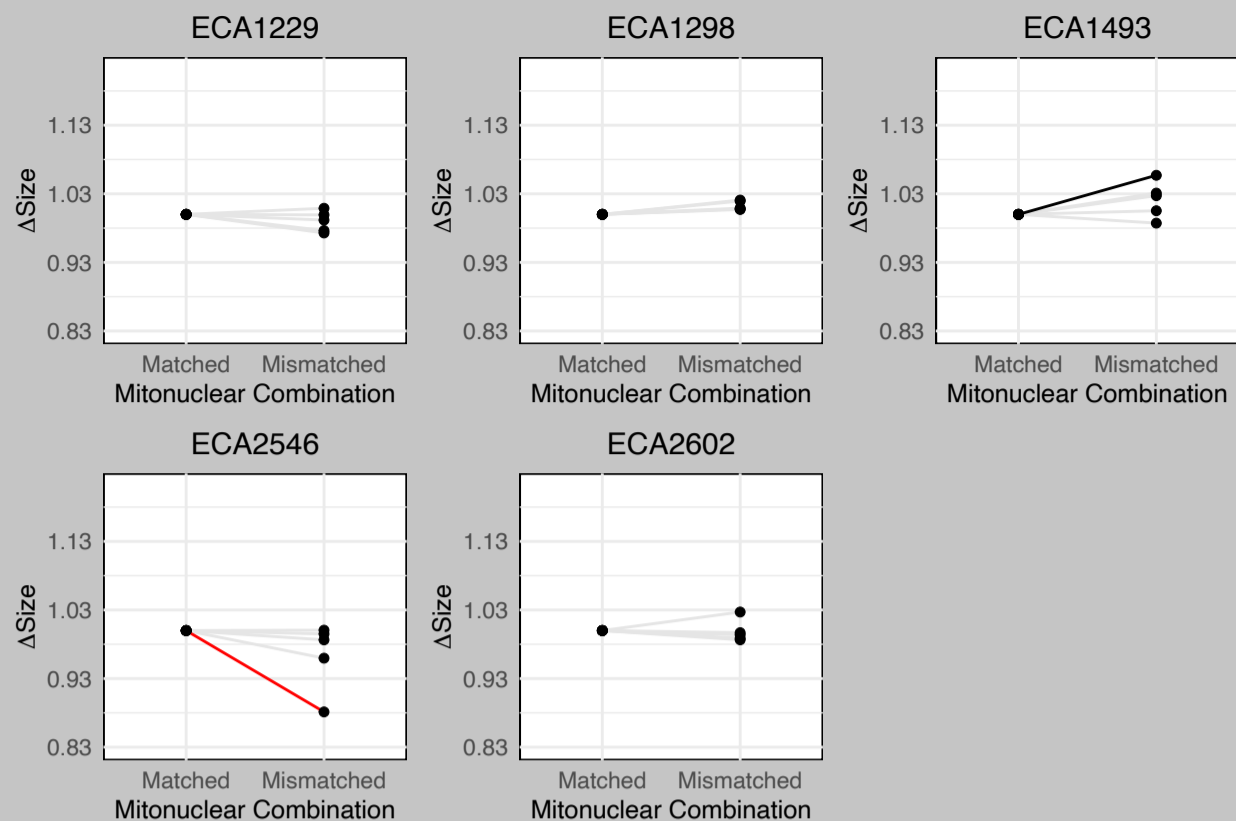

25°C

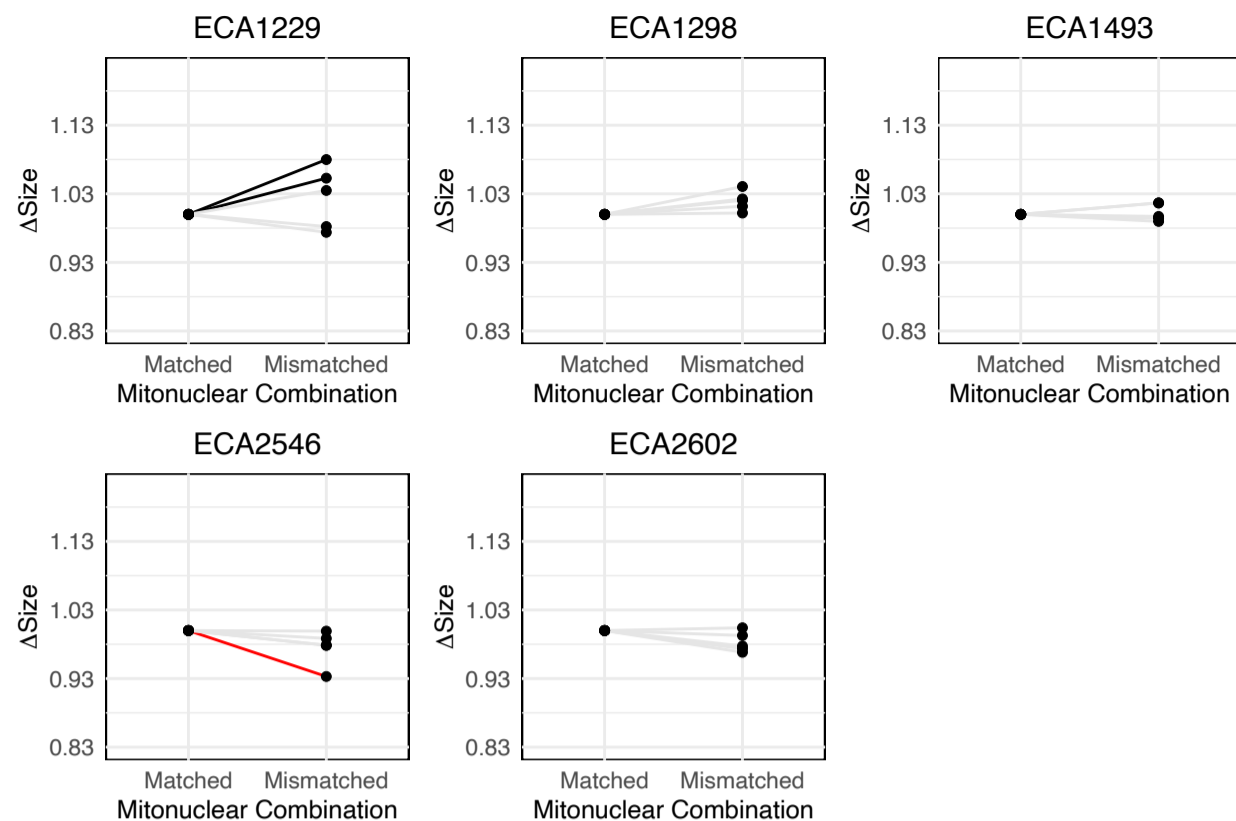CuCl<sub>2</sub> [75 uM]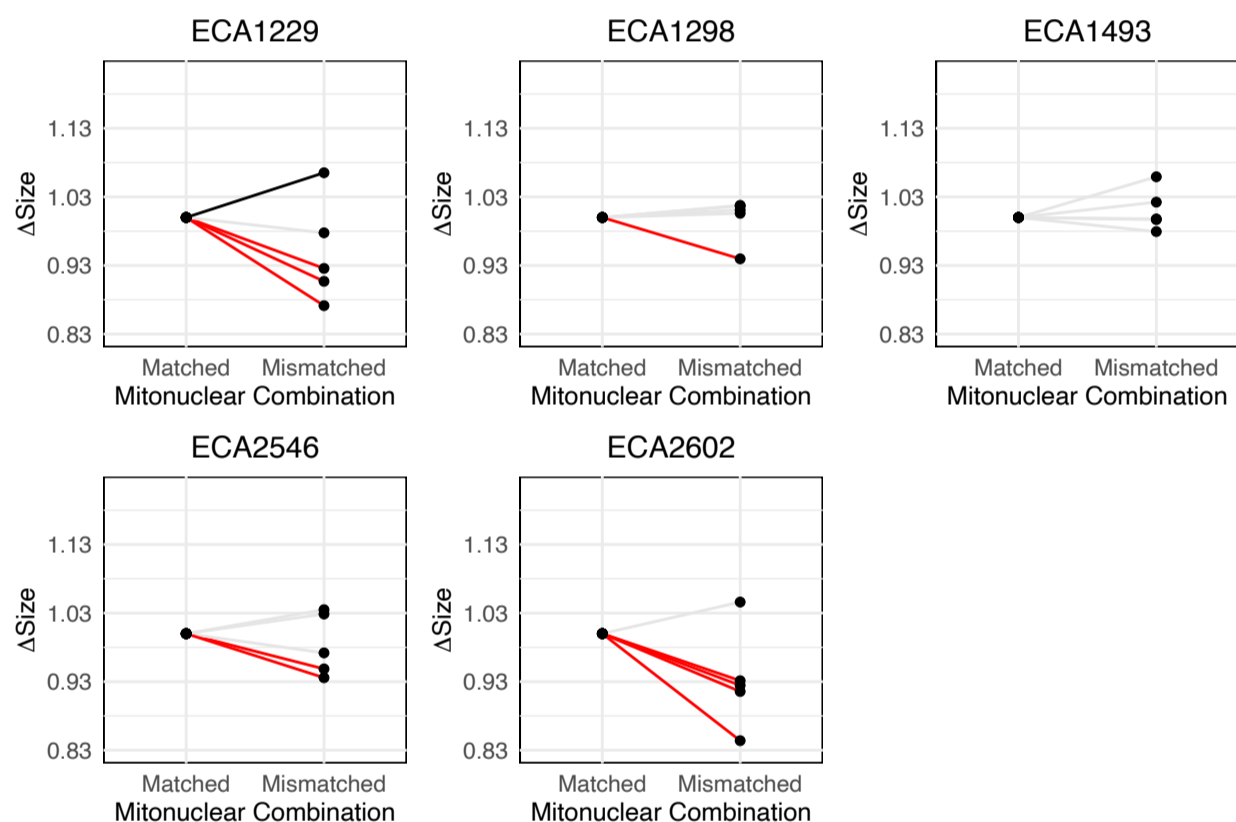NiCl<sub>2</sub> [75 uM]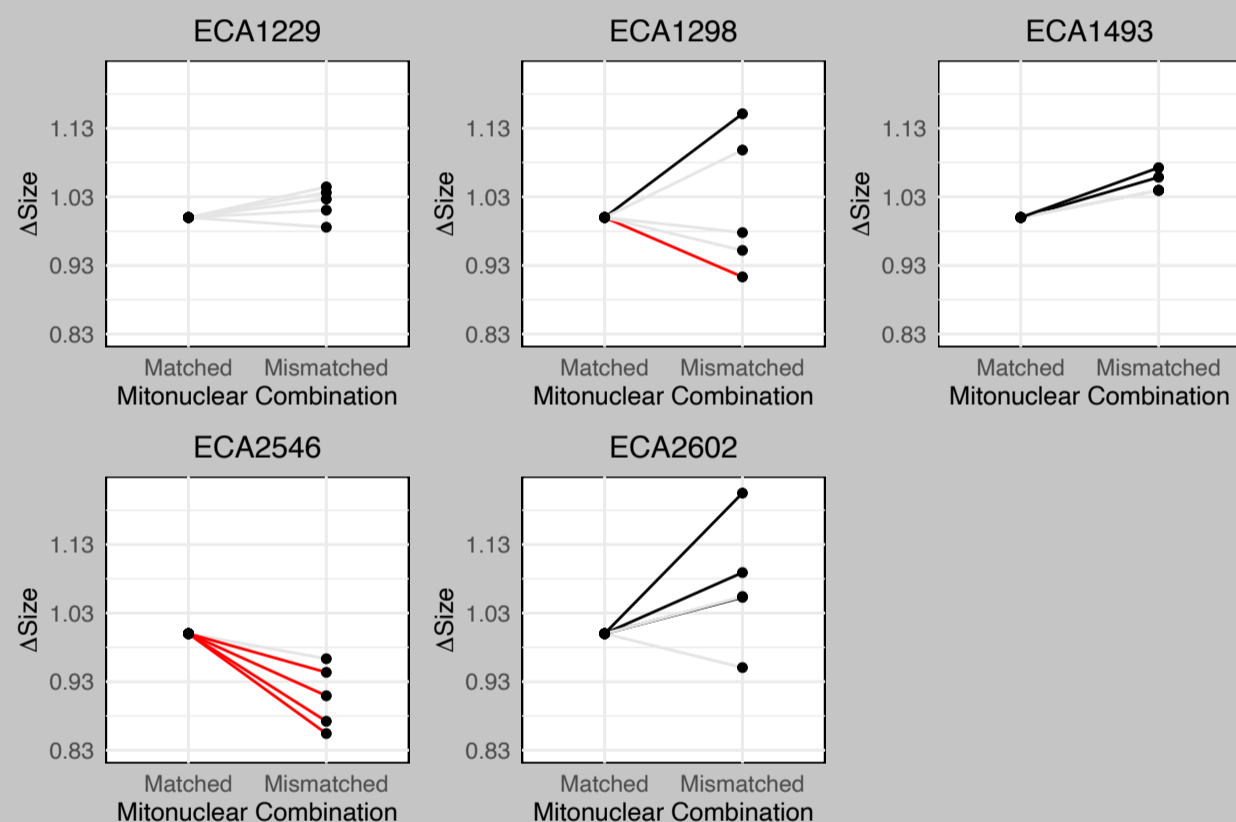

Paraquat [100 uM]

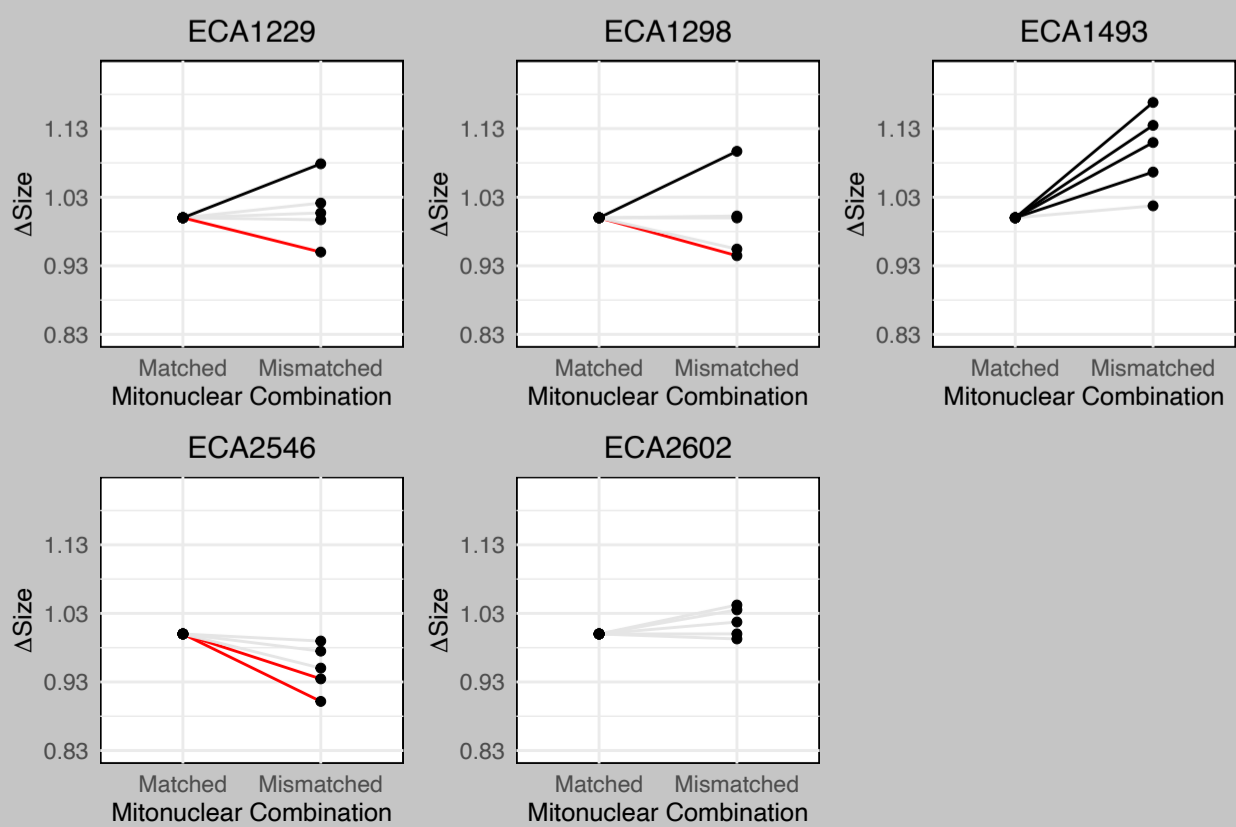ZnSO<sub>4</sub> [100 uM]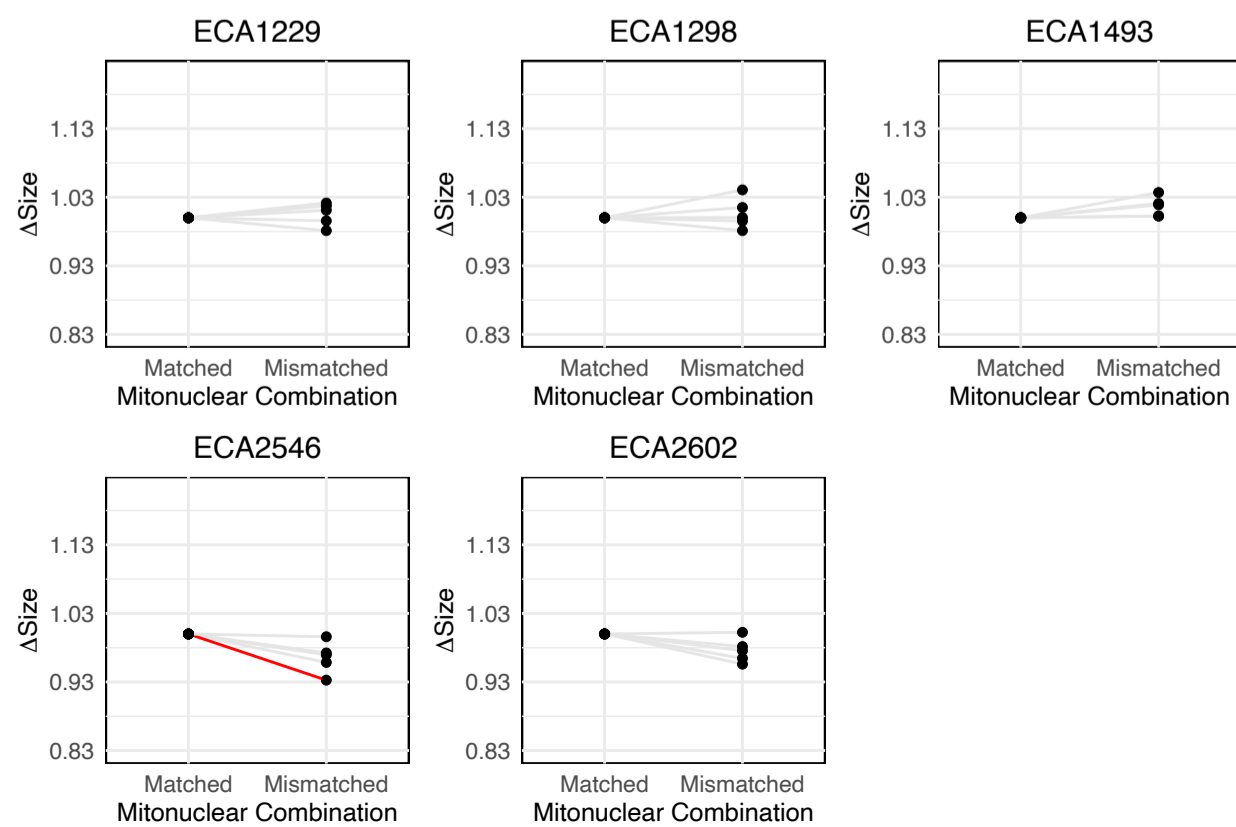

Supplement: msaf300_Supplementary_Data [file msaf300_supplementary_data.zip › Figure S6.pdf]
